# *In vitro* genotoxicity testing strategy for nanomaterials and the adaptation of current OECD guidelines

**DOI:** 10.1016/j.mrgentox.2011.09.013

**Published:** 2012-06-14

**Authors:** S.H. Doak, B. Manshian, G.J.S. Jenkins, N. Singh

**Affiliations:** Institute of Life Science, College of Medicine, Swansea University, Singleton Park, Swansea SA2 8PP, Wales, UK

**Keywords:** Nanoparticles, Nanotubes, Nanomaterials, Genotoxicity, Micronucleus assay, Ames test

## Abstract

There is a pressing requirement to define a hazard identification and risk management strategy for nanomaterials due to the rapid growth in the nanotechnology industry and their promise of life-style revolutions through the development of wide-ranging nano-containing consumer products. Consequently, a battery of well defined and appropriate *in vitro* assays to assess a number of genotoxicity endpoints is required to minimise extensive and costly *in vivo* testing. However, the validity of the established protocols in current OECD recognised genotoxicity assays for nanomaterials is currently being questioned. In this report, we therefore consider the *in vitro* OECD genotoxicity test battery including the Ames, micronucleus and HPRT forward mutation assays, and their potential role in the safety assessment of nanomaterial induced DNA damage *in vitro*.

## Highlights

► We consider current *in vitro* OECD genotoxicity tests for nanomaterials. ► Ames test does not appear to be suitable for nanomaterial assessment. ► *In vitro* HPRT and micronucleus assays require nanomaterial specific protocols. ► We recommend a strategic *in vitro* genotoxicity testing strategy for nanomaterials.

## 1 Introduction

The dramatic expansion in the nanotechnology industry over the last decade has resulted in the development of a myriad of novel materials specifically in the nano-size range (sub-100 nm). These nanomaterials (NM) are promising to revolutionise our life-style as they have unique physico-chemical features encompassing beneficial properties surpassing those of traditional substances. Such features include enhanced electrical or thermal conductivity, more efficient catalysts, high tensile strength (yet lighter weight) or improved drug delivery vehicles. Consequently, NM have potential applications in a wide range of industrial settings in addition to medical healthcare and consumer products. Indeed there are over 1000 products in the consumer market-place that include NM (Woodrow Wilson Database; www.nanotechproject.org) and this is projected to substantially increase in the near future with some estimates indicating a 19% predicted growth of the global nanotechnology industry between 2011 and 2013 [1,2]. Thus, the need to define a hazard identification and risk management strategy for these new products is of increasing importance and is a hotly debated topic.

Regulating new technologies requires a fine balance as industry and consumers drive the desire for novel products to maintain a competitive edge and enhanced life-style, respectively. Consequently, whereas a fast-track through the regulatory process is desirable, inappropriate over-regulation can potentially damage the nanotechnology industry, limiting future innovation and international competitiveness [3]. Clearly a delicate balance is required to prevent stifling innovation while ensuring that public and environmental safety are protected.

An important aspect of hazard identification includes the potential for a novel agent to induce genotoxicity, as damage to the genetic material may result in the induction or promotion of carcinogenesis, in addition to reproductive impacts if germ cell DNA is compromised. The mechanisms likely to be responsible for NM induced genotoxicity fall into two main categories, namely primary and secondary mechanisms. Primary mechanisms are those imparted by the NM themselves at the level of the single-cell and may be either the result of direct or indirect interaction between the NM and DNA (and/or its regulatory apparatus). Direct acting agents are those that are DNA reactive i.e. they come into direct contact with the genetic material causing physical or chemical damage. In contrast, indirect acting agents induce genotoxicity by damaging intermediate biomolecules that are often components of the cell division machinery and their subsequent malfunction causes DNA aberrations. Alternatively, indirect DNA damage may arise as a result of the induction of intermediates, e.g. in the case of oxidative stress, where NM exposure results in the excessive generation of reactive oxygen species (ROS) that are responsible for damaging biomolecules including lipids, proteins and DNA. The secondary mechanism for genotoxicity refers to the ability of a NM to induce a (chronic) inflammatory response *in vivo* that results in excessive generation of ROS by macrophage and neutrophil cells recruited to the exposure site as a defence mechanism. Normally, acute inflammation enables the removal of foreign bodies by the immune cells, but if the immune system is unable to cope with the clearance of NM, or is compromised in some manner, then the result is chronic inflammation, promoting oxidative stress that is subsequently responsible for damaging surrounding cells within the tissue. Distinguishing between the production of ROS as a primary indirect DNA damage mechanism of the NM itself and its generation as part of a secondary inflammatory reaction is difficult but can be achieved through manipulation of testing protocols. The former can only be detected in 2D *in vitro* culture systems utilising non-immune cell types, as any ROS detected in this situation is not imparted by inflammatory cells and therefore must be a consequence of the NM itself. In contrast, to detect oxidative stress induced as part of a secondary inflammatory response, *in vivo* testing would be required to establish if chronic inflammation is induced following exposure to the NM, together with simultaneous detection of elevated oxidative stress and associated DNA lesions. This would not completely rule out primary mechanisms for genotoxicity through indirect routes for the generation of oxidative stress, but would be a strong indicator for the role of a secondary inflammatory genotoxicity mechanism (particularly if a coupled *in vitro* assay in non-immune cells was negative for oxidative stress).

Due to the variety of mechanisms leading to NM induced DNA damage and the range of mutagenic events that may occur as a result, a battery of testing systems is required to establish the genotoxic potential of a substance under investigation. For pharmaceutical and chemical compounds, a battery of well defined tests to assess a number of genotoxicity endpoints (point mutations, aneuploidy and chromosomal fragmentation) is necessary for regulatory approval. The current test guidelines largely include the *in vitro* bacterial reverse gene mutation test (Ames; OECD 471), an *in vitro* mammalian cell gene mutation test (e.g., HPRT forward mutation assay, mouse lymphoma TK assay; OECD 476) and an *in vitro* mammalian cell chromosome aberration or micronucleus assay (OECD 473 or 487, respectively) [4]. However, it has recently been suggested that the *in vitro* testing regime for chemicals needs modification in order to minimise false positive results and thereby reduce subsequent *in vivo* testing. Consequently, Kirkland et al. [5] conducted a detailed study in which they demonstrated that performing the Ames test and *in vitro* micronucleus assay was ample for the identification of *in vivo* genotoxins and carcinogens, detecting 78% of *in vivo* genotoxins (the remainder being either negative, weak or inconclusive *in vivo* genotoxins). Despite the debate that exists around the best testing regimen to minimise costly *in vivo* experimentation, the regulatory requirements for chemical compounds are clear and all assays required for genotoxicity testing have definitive OECD guideline protocols that are strictly adhered to. In contrast, testing of NM or nano-containing products for genotoxicity is a grey area that has yet to be well defined and the best battery of tests for hazard identification is currently unknown.

The question that remains is whether or not the current regulatory testing regime and protocols are suitable for NM? There has been much debate over whether NM can be treated in the same way as chemical or pharmaceutical products, or if they require their own specific testing protocols because of their unique physico-chemical properties. Indeed, the evidence accumulating demonstrates that NM interact with a range of assay components including those that are fundamental to genotoxicity tests, thus highlighting the importance of generating well validated assay protocols for NM specifically [6,7]. In this report, we will consider the current *in vitro* OECD test battery for genotoxicity and their potential role in the assessment of NM induced DNA damage.

## 2 Bacterial reverse mutation test (OECD 471)

The Ames test is the bacterial reverse mutation test used to determine the mutagenicity of exogenous substances. The test identifies mutagenic compounds as those capable of reverting point mutations in histidine or tryptophan biosynthesis genes in *Salmonella typhimurium* or *Escherichia coli*, respectively, restoring the ability of the bacteria to generate these essential amino acids. Usually, a combination of 5 *S. typhimurium* strains, or 4 *S. typhimurium* strains plus 1 or 2 *E. coli* strains are required to detect a range of base substitution or frameshift events. The ease and cost effectiveness of the test system make it widely used in the safety analysis of chemical substances. It is an essential test within the current battery of assays required for genotoxicity evaluation and has also recently been highlighted as one of the two assays recommended by the UK expert advisory Committee on Mutagenicity (and [5]) that in parallel have sufficient sensitivity to detect carcinogens and *in vivo* genotoxins while minimising the risk of false positive *in vitro* genotoxicity testing results.

Although this test has proven to be invaluable in the safety testing of chemical substances it has been less commonly used with NM. To date, 19 studies have been published where the Ames test was utilised for the genotoxicological analysis of NM. Seventeen of these studies reported negative mutagenicity in the Ames test [8–24]. The remaining two studies only reported weak mutagenic effects with water-soluble iron–platinum (FePt) nanoparticles capped with tetramethylammonium hydroxide [12] and zinc oxide (ZnO) and titanium dioxide (TiO_2_) nanoparticles [25]. Interestingly, even though many nanomaterials are negative for mutagenicity in the Ames test, they have largely been found to have positive genotoxic responses in other *in vitro* mammalian cell test systems (Table 1) including the chromosomal aberration [9,19,26–29], micronucleus [30–40] and comet assays [11,30,34,41–45], in addition to positive *in vivo* test results [13,32,34,46–49].

It can be argued that the discrepancies between the Ames test and other genotoxicity assays highlighted are due to treatment dose, the choice of NM dispersant and slight differences in the physico-chemical characteristics of the NM under study. However, in some cases different genotoxic responses with the Ames test and other tests are observed within the same report. For example, Dufour and colleagues found zinc oxide nanoparticles were non-mutagenic in the Ames test yet largely clastogenic *in vitro* in Chinese hamster ovary (CHO) cells in their chromosomal aberration studies [9]. Another genotoxicity investigation by Kisin and colleagues [11] found similar differences with single walled carbon nanotubes (SWCNT) using the Ames, comet and micronucleus assays. Micronucleus assay results indicated some significance in chromosomal damage in V79 cells and the comet assay demonstrated an increase in DNA damage after only 3 h of SWCNT exposure. However, exposing two different strains of *S. typhimurium* (YG1024 and YG1029; TA98 and TA100 derivatives, respectively) to the SWCNT gave rise to negative results for mutagenicity [11]. A final example is from work conducted by Balasubramanyam and colleagues in a series of publications on aluminium oxide nanoparticles. These nanoparticles caused size- and dose-dependent genotoxicity in the *in vitro* micronucleus assay [36] and *in vivo* comet and micronucleus assays following oral exposure to rats [29,34]. However, they were negative in the Ames test based on *S. typhimurium* TA97a, TA98, TA100, TA102 and TA1535 strains [21]. It should be noted that the Ames test is poor at detecting genotoxins that induce large-scale DNA damage. This may therefore indicate that the NM generate large deletions that are detectable in tests such as the micronucleus or chromosome aberration assays, but cause a negative Ames result through deletion of the histidine gene, which negates reversion of its defect and leads to cell death. However, this would suggest that the Ames test would demonstrate comparatively higher levels of cytotoxicity, which is not observed.

The studies described consequently indicate that although the Ames test is a reliable genotoxicity screen for the analysis of chemicals, it does not appear to be suitable for the assessment of NM. This might be related to the degree of NM uptake by the bacterial cells, which is likely to be less than in human cells for two reasons. Firstly, prokaryotes cannot perform endocytosis and secondly, their cell wall forms a barrier against simple diffusion of NM (particularly those in agglomerated from) into the bacterial cell – this lack of uptake could potentially lead to false negative results. It is however, possible that very well dispersed NM of approximately 20 nm diameter could enter the bacterium and therefore the system would report genotoxicity appropriately. Additionally, if NM induce DNA damage as a consequence of ion release then those ions would also penetrate the bacterial cell wall. These scenarios may therefore account for some of the weak positive results observed in the literature to date.

Finally, it is also important to consider the fact that some NM harbour antimicrobial activity. For example, Tran and colleagues reported that iron oxide nanoparticles inhibited the growth of *Staphylococcus aureus* bacteria [50] and nanosilver is also a well recognised antibacterial agent [44]. Thus, the Ames test is unlikely to be a suitable general *in vitro* genotoxicity test for NM but at the moment the data on its use is limited and so it cannot be completely ruled out at this stage without a co-ordinated research program directly comparing its response to other *in vitro* and *in vivo* tests in parallel. Additionally, modifications to the technique may need to be considered to promote uptake of NM into the Ames test bacteria to reduce the potential for false negative results.

## 3 *In vitro* micronucleus assay (MNvit OECD 487)

**Fig. 1:**
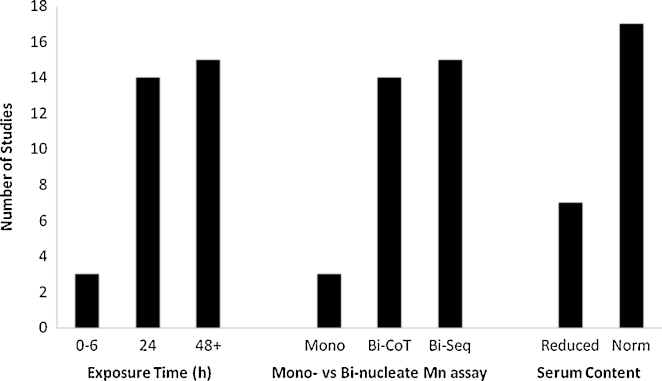
Variation in micronucleus assay protocol used in studies investigating NM genotoxicity. Mono: mononucleate micronucleus assay; Bi-CoT: binucleate micronucleus assay with co-treatment of MN with cytochalasin-B; Bi-Seq: binucleate micronucleus assay with sequential treatment of NM followed by cytochalasin-B. Note that the overall numbers in each category are not equal as multiple conditions were tested within the same studies. Data collated from [38,51,60,88].

The *in vitro* micronucleus assay (MNvit) rapidly determines the frequency of gross chromosomal damage induced by a test agent. It has gained popularity as the test of choice over the chromosome aberration assay because it is substantially quicker to perform, easier to analyse and readily detects aneugens as well as clastogens (which the chromosome aberration assay cannot do unless it is specifically modified). Thus, the MNvit is now recommended as one of the two core *in vitro* test systems to characterise the genotoxicity of chemical and pharmaceutical agents [5]. However, methodological variations in the assay that have no impact when studying chemical compounds, can dramatically affect the data-sets when testing NM [6,51]. A recent review by Gonzalez et al. [51] summarised all the studies that have utilised MNvit between 2002 and 2010 and the variations in the protocols employed. This information together with several more recent publications that have since gone to press is illustrated in Fig. 1. As can be seen, there is considerable variation in the methodologies selected to date with most studies opting for 24 h + exposure times, full serum content in exposure media and the binucleate version of the micronucleus assay, although the selection between co-exposure of NM with cytochalasin-B and separation of those two steps is almost equal. Another factor not illustrated here is the large variation in cell lines selected for the studies.

Currently, the influence of each of these protocols on variability in resultant data-sets is unclear, but several observations are making it apparent that we need to have certain criteria in place to work towards standardising the methodology for NM. For example, cytochalasin-B has been shown to prevent the cellular uptake of NM in some cases due to its capacity to interfere with formation of the actin-filaments required for endocytosis [6]. Thus, it is important that when using the binucleate version of the MNvit, co-exposure studies where NM are exposed to the test cells at the same time as the cytochalasin-B are avoided. Instead, cells should be dosed with the NM and the cytochalasin-B applied as a separate step at a later time point. It should be noted that this provides the option for post-treatment with cytochalasin-B (i.e. application of NM for a given period of time, then removal of the NM and replacement with media containing cytochalasin-B) or delayed co-treatment (i.e. application of NM for 3–24 h, followed by the addition of cytochalasin-B for the remainder of the treatment time), but which of these procedures is preferable has not been validated yet and requires further investigation [6,51]. Few studies have examined the micronucleus frequency induced at different exposure time-points for the same NM in parallel, but interestingly those that have performed such investigations demonstrate that 24 h exposure (on average equating to 1.5–2 cell cycles, as required by the OECD 487 guideline) results in slightly higher micronucleus frequency or lower positive doses than shorter or longer dosing durations [52,31]. Thus, suggesting a 24 h exposure is ample to determine genotoxic response, as it allows the cells to go through more than one complete cell cycle, which is important given that the NM may come into direct contact with the DNA when the nuclear membrane breaks down during mitosis. Furthermore, it is likely that the time required for endocytosis to be completed will be important and is NM dependent, thus a cautionary approach might be to allow a 24 h exposure prior to addition of cytochalasin-B.

Another aspect to consider is the serum content included in the culture media during exposure. As illustrated in Fig. 1, approximately 2/3 of the MNvit studies performed to date have used a full serum complement in their media. However, several studies have previously demonstrated that reducing serum content can enhance or reduce NM cellular uptake in a cell type- and NM-dependent manner, which in turn will affect the level of intracellular damage induced [6,53–55]. Until we have a better understanding of the factors that govern cellular uptake with specific regard to serum content, it is therefore advisable that genotoxicity studies consider in parallel both complete and reduced serum content in their media and report any consequent differences that may be observed.

Finally, extension of the MNvit to include kinetochore or centromeric staining enables discrimination between aneugenic (chromosome loss or gain) or clastogenic (chromosome fragmentation) events [56]. This provides important additional information on the origin of the micronuclei induced, and thus some insight into the DNA damage mechanism that may have been imparted by the NM exposure. This simple experimental addition is often overlooked by investigators using the MNvit, but its inclusion is highly recommended to enhance our mechanistic understanding of NM induced damage, as opposed to maintaining the assumption that all impairments are a consequence of oxidative stress.

## 4 HPRT forward mutation assay (OECD 476)

Given that there are potential issues with the use of the current bacterial mutagenicity testing system (Ames) as described in Section 2, it is prudent to utilise a mammalian cell alternative for assessing mutagenicity. This is particularly important, as no single assay is capable of detecting all forms of DNA damage (mutation and chromosome damage in particular). Several such tests exist, but the most frequently applied examples are the mouse lymphoma assay (MLA), which utilises the autosomal thymidine kinase (Tk) gene as a reporter of mutations in the L5178Y/*Tk*^*+/−*^ mouse lymphoma cell line; and the X-chromosome linked hemizygous hypoxanthine guanine phosphoribosyltransferase (HPRT) gene forward mutation assay. An advantage of both of these assays over the Ames test is the capacity to characterise the diverse range of possible mutations induced through sequencing the mutated Tk or Hprt loci, thereby increasing our understanding of the mutational origin. The MLA only utilises the L5178Y rodent cell line, and although rodent cells are often used for the HPRT assay (e.g. L5178Y mouse lymphoma cells, Chinese Hamster Ovary Cells, V79), an important advantage of this test system over both the Ames test and MLA is that it can also be applied to a range of human monolayer or suspension cell types provided they are male (such as TK6 or MCL-5). At present, there are no reports in the literature that have assessed NM mutagenicity with the MLA, therefore genotoxicity data based on this test system is particularly lacking. In contrast however, the HPRT assay appears to have been a more popular choice for NM mutagenicity studies, but it is important to note that even these investigations are still very low in number.

Wang and colleagues [31,57,58] have utilised the HPRT assay to demonstrate ultra-fine silica and quartz are able to induce point mutations in WIL2-NS, a human B-cell lymphoblastoid lineage. However, it should be noted that WIL2-NS cells are more sensitive to induced mutations are thus are more mutable than other lymphoblastoid cell lines (e.g. TK6) due to a p53 deficiency and comparatively high DNA recombination phenotype [59]. Interestingly, the role of inflammatory cells in quartz-associated mutagenesis have been highlighted by Driscoll et al., who demonstrated a significantly increased HPRT mutation frequency in rat lung epithelial cells (RLE-6TN) following their co-culture with bronchoalveolar lavage fluid (BAL; containing particle-elicited inflammatory cells) extracted from quartz-exposed rats [60]. This investigation therefore suggests a secondary mechanism of action involving inflammatory cells or intermediates (in the BAL fluid) were responsible for the observed genotoxicity. Furthermore, inclusion of catalase in the co-culture attenuated the mutagenic effect of the BAL cells, thus providing evidence for the potential role of ROS as a by-product of inflammation in eliciting the observed mutagenic activity.

As with many nanotoxicity studies in the literature, there is some conflict in those that have utilised the HPRT assay, with some detecting significant levels of mutagenicity induced by TiO_2_ and carbon nanotubes [37,57], while others report no induction of HPRT mutants with these NM [61,62]. It is most likely that these conflicts are due to slight differences in the physico-chemical characteristics of the NM, but interestingly, the studies negative for HPRT mutants utilised rodent cell lines as opposed to human cells in the positive investigations. It is therefore possible that different sensitivities of the cell lines used may also play a role in the discrepancies observed. Of importance is the fact that these variations in cell line sensitivity may not just be due to the species of origin, but also the relative fidelity of DNA repair capacity in the cells selected. If cells with reduced repair capacity are utilised for the HPRT assay (e.g. WIL2-NS) then false positive data-sets could potentially be the result. Thus, careful consideration of the cell lines used for the *in vitro* HPRT assay is necessary.

HPRT studies following current OECD guidelines are routinely conducted for NM within our laboratory without any notable issues. However, one observation has been that some NM cannot be effectively washed off cells (whether suspension or monolayer), thus the true exposure period may be longer than the actual dosing time which might lead to higher levels of damage. It is also advisable to avoid the freeze-thaw procedure sometimes utilised after exposure to stagger plating in selective media (used to make large dose-series more manageable), as the exposed cells appear to exhibit delayed or no cell revival or proliferation. This could be due to significant internalization of the NM under study, which could have an impact on cellular processes such as replication, transcription and translation [63].

At present it is difficult to truly evaluate the *in vitro* HPRT forward mutation assay for reliability in detecting NM that are mutagens as no parallel *in vitro* versus *in vivo* mutagenicity studies have been performed. However, in the absence of such investigations it is prudent to follow OECD guidelines for this assay.

## 5 Assays of the future

The field of genotoxic hazard identification is constantly evolving and test systems are continuously being modified and improved through rigorous scientific efforts and international collaborative studies comparing slight variations in test protocols. These modifications can make existing tests both more sensitive and more specific. Examples include the refinement of the appropriate cell lines for use in specific tests (e.g. p53 competent cells) as well as consensus agreements on appropriate dose ranges to remove the use of irrelevant “high doses” of test agent [64,65]. Obviously in the emerging field of nano-genotoxicology, assay refinement is also vital and will undoubtedly play a major role in developing testing strategies tailored to optimally detect NM genotoxicity.

However, it is important to also consider that the current genotoxicity testing battery for chemicals has evolved over the past 3 decades and continues to evolve with new tests appearing in recent years (e.g. *in vitro* micronucleus assay) and some older tests being largely discarded. New tests developed to identify hazards and assess risks of mutation in the chemical arena may also prove to be extremely useful in NM testing. A relatively new high-throughput test system that has shown much promise in testing chemicals for genotoxic activity is the GADD45 (GreenScreen) assay [66]. This assay is a fluorescent reporter-based system that operates in a wide range of cell backgrounds and is built on the observation that up-regulation of the GADD45 gene is a sensitive surrogate marker of most types of DNA damage. Hence a fluorescent reporter construct is used in conjunction with a plate reader, with post-exposure cellular fluorescence being measured to indicate DNA damage. Previously, there has been very good validation of the link between GADD45 expression and genotoxicity [67], but no studies to date have utilised this type of reporter system to study NM induced genotoxicity. Its main benefit may lie in its high-throughput nature, enabling multiple physico-chemical NM variants to be tested in parallel to determine which parameters are important in determining genotoxic potential.

Another new and exciting genotoxicity test that is gaining widespread momentum in the field currently is the PIGa assay [68]. The PIGa gene (which is x-chromosome linked and hence has only 1 copy in males and only 1 functional copy in females) codes for a protein (phosphatidylinositol glycan complementation group A) heavily involved in the glycosylphophatidylinositol (GPI) anchors that attach cell markers to the cell membrane of blood cells. The PIGa mutation assay has been developed to identify cells that have lost these specific cell surface markers (e.g. CD59) through mutation of the PIGa gene, leading to their loss of expression in the cell membrane. Flow cytometry is used in conjunction with fluorescent antibodies for the cell markers of interest, to screen through tens/hundreds of thousands of cells in search of the relatively rare cells without expression of the cell marker. These are identified as the cells with PIGa mutations and frequency of these cells informs on the mutation frequency induced by exposure to exogenous agents. It has been suggested that this mutation is neutral (neither harmful nor advantageous) and hence mutant cells may accumulate with time and not be removed, making them easier to identify. This assay is currently in development and initial comparative studies show very promising results for chemical genotoxins. The advantage of this kind of system for genotoxicity in general, but also for NM induced genotoxicity is the fact that the same approach can be used *in vitro* for hazard identification and *in vivo* in animal models to perform risk assessment. Furthermore, as human and animal cells can both be studied with this approach, then even biomonitoring of workers exposed to NM during manufacture and processing is feasible with this system.

## 6 General experimental considerations

In addition to test system selection, there are a number of factors that require some consideration to minimise false positive or negative results in the battery of *in vitro* genotoxicity assays [6,69]. A critical issue is dose selection – concentration ranges selected should be related to physiological relevance. Many studies use excessively high exposures that may generate a genotoxic response, but bear little resemblance to true exposure levels that may be experienced in the environment or by human populations. However, a complication is that a low exposure to NM could accumulate in cells over time, thus representing a higher cumulative dose, a situation that is difficult to mimic in acute *in vitro* tests. Furthermore, for many NM, consumer applications are still in development and consequently our understanding of exposure levels is limited. It is therefore important at this time that detailed dose–response data-sets are generated and where applicable, maximum cumulative exposure information indicated with concentrations 10-fold above and below this point to ensure that the data is still of value as our understanding of dose accumulation and true cellular exposure develops.

Selection of cell lines for the *in vitro* genotoxicity assays is also a source of conflict highlighted by a recent study where 10 cell lines yielded different cytotoxicity responses to 23 NM due to variations in their sensitivity [70]. However, it must be noted that this is also a burning issue in chemical genotoxicity and extensive evaluation has been underway for some years to identify the cell lines that minimise false positive results [64]. An additional consideration with NM is the different exposures that adherent cells versus suspension cells are subjected to due to the gravitational settling of heavier agglomerates as highlighted previously [6] and the importance of tissue related exposure scenarios as a basis for selection of cell types for study have also been stressed [69,71]. For genotoxicity assessment specifically, a critical factor is the karyotypic stability of the cell line – this must be ensured particularly where cells derived from cancer lineages are utilised as they may be more or less sensitive to DNA damage responses if they are genetically compromised. Thus, in the absence of exposure data or genetically stable cell lines, those cells highlighted by the COLIPA project to minimise false positive results in genotoxicity assays are recommended as a basis for defining the genotoxic capacity of the NM under study [64].

In addition to the *in vitro* genotoxicity data generated by the above testing systems, a vital aspect of NM safety assessment is a detailed characterisation of the physico-chemical features of the test material. This is necessary as it is now well accepted that subtle differences these characteristics can dramatically affect biological response. Much of the early nanotoxicology-based publications report numerous conflicting results, but these apparent differences are most likely due to slight variations in the physico-chemical features of the materials under study. For example, titanium dioxide can exist in either a rutile or anatase crystalline form, which behave very differently from one another in biological systems with the rutile form inducing more pulmonary toxicity than the anatase form [13]. Thus, if this detail on the crystalline structure were omitted from the description of the material, the consequence would be an apparent conflict in data-sets.

Numerous studies have demonstrated size-dependent toxicity including mitochondrial damage, oxidative stress, chromosomal and oxidative DNA damage, usually with smaller sized NM causing significantly more damage than their larger counterparts [72–74]. Additionally, surface charge dependent cellular uptake and toxicity has been shown by some groups, with cationic nanoparticles being more toxic than their anionic counterparts [75–78]. Other investigations have concluded surface coating plays an important role in NM-induced cellular damage as substituting one ligand for another can modulate toxicity. For example Takahashi et al., has shown that replacing cetyl trimethylammonium bromide (CTAB) with polyethylene glycol (PEG) on gold nanorods results in reduced toxicity [73,79,80]. Yet, depending on the degree of exposure and rate of clearance, PEGylation can sometimes cause organ (particularly renal) toxicity, albeit at high doses [81]. Furthermore, NM are being considered as vehicles for drug delivery and thus in these cases testing of the combined package to be distributed is also important. Therefore, well-thought experimentation needs to be considered and conducted to delineate the root-cause of any adverse cellular effects observed.

Another important physicochemical characteristic, which is more relevant to studies involving metal nanoparticles is the need to distinguish between the toxic effects of metal/metal oxide nanoparticles and their corresponding dissolved metal ions. Some of the common metal ions that can be released from the core nanoparticles upon corrosion (or particle dissolution) over time include, Zn^2+^, Co^2+^, Ag^+^ and Fe^3+^[82–85]. While the metal/metal oxide nanoparticles may exhibit one set of toxicological responses, the metal ions may promote an altogether different, overlapping or similar set of cellular damage endpoints [82–84]. These are just a small number of examples as details on the physico-chemical characterisation required have been previously described [86] and are an essential component that must accompany any nano(geno)toxicological assessment.

## 7 Future considerations

The *in vitro* genotoxicity assessment framework for NM still requires some validation based on firm experimental evidence that is currently lacking, hence the difficulty in our ability to define the regulation required for these new products. Some of the key issues that require clarification at present include:

1. Assessment of which *in vitro* assays best correlate with those results obtained from *in vivo* studies using identical NM in the test systems performed in parallel.
2. Determination of which *in vitro* tests are the most informative, reducing false positive/negative results with NM to minimise subsequent *in vivo* experimentation (e.g., is the Ames test suitable for NM genotoxicity testing). It is not necessary to perform all assays, but we must be able to determine what the most appropriate battery should consist of and what assay modifications are required for NM testing.
3. Cell line selection – will the use of suspension versus adherent cell lines heavily influence the results? Do the cell lines need to represent the target tissues associated with exposure, or will those highlighted by the COLIPA project suffice?
4. Degradation of NM in test systems is something that is still very poorly understood. Does this mimic what happens *in vivo*? Do assay components aid degradation (e.g. DMSO)? Are the degradation products more problematic than the NM themselves and if so can this be used to develop acellular screening assays prior to *in vitro* testing?
5. Current *in vitro* assays do not take into consideration the consequences of chronic exposures, when cumulative effects over time may be more detrimental than those resulting from acute exposures. This is particularly pertinent for biopersistent engineered NM, where associated adverse cellular effects may not be apparent in short-term acute assays.
6. New *in vitro* assays are required that are able to report on DNA damage induced by secondary mechanisms involving the promotion of chronic inflammation (e.g., assays focusing on cytokine release or novel co-culture methods with inflammatory cells).

Currently, the validity of *in vitro* testing systems for NM has come into question, largely because of our limited understanding of their pharmacokinetics including absorption, distribution, metabolism, excretion (ADME) following the wide ranging NM exposure scenarios envisaged. Additionally, traditional *in vitro* genotoxicity assays only report on primary DNA damage, thus secondary mechanisms for damage involving inflammatory responses require *in vivo* studies [87]. However, well validated *in vitro* assays present a number of advantages over *in vivo* testing regimens such as their reduced cost, ability to screen large numbers of test materials relatively quickly, and capacity to establish mechanisms of action. Further, the obvious ethical issues surrounding the 3Rs (reducing, replacing and refining animal usage) must be taken into consideration to minimise the burden on animal testing. Additionally, if NM are in cosmetic products then the only option in Europe is *in vitro* testing as a result of the ban on *in vivo* genotoxicity testing in this industry (7th Amendment to the EU Cosmetics Directive). Thus, the validation of current *in vitro* assays, establishment of suitable *in vitro* test batteries to cover a range of applicable end points, and the development of new *in vitro* tests (perhaps based on co-culture or 3D model systems) is becoming ever more important and warrants significant attention.

## 8 Conclusions

A strategic approach is clearly required to answer the remaining questions with regards to the genotoxic *in vitro* testing strategy required for the safety assessment and regulation of NM, but in the interim it is recommended that the following approach be taken:

• Complete description of the physico-chemical features of the material under investigation;
• Mammalian cell chromosome aberration test (if this is to be the micronucleus assay, then the appropriate adaptations described above should be considered);
• Mammalian cell point mutation test (e.g. HPRT assay);
• Extended dose responses are required, which is important given true exposure concentrations are unknown at present and likely to change over the coming years as new commercial applications are developed and marketed.

This strategy involves the use of test systems that analyse different DNA damage endpoints (chromosomal aberrations and point mutation), which at this early stage in the life of Nano Genotoxicology is an important consideration given our limited understanding of the spectrum of genotoxic mechanisms involved. Indeed there is evidence to suggest that NM can impart different types of DNA aberrations [37]. Such a strategy could therefore form a first-stage *in vitro* assessment to screen NM for further *in vivo* genotoxicity evaluation.

## Conflicts of interest statement

All authors declare that they have no conflicts of interest.

## Figures and Tables

**Table 1:** Summary of some nanomaterials studied to date for DNA damage in commonly used *in vitro* and *in vivo* genotoxicity test systems. Black dot presents positive genotoxic results while the empty circles indicate negative results.

Nanoparticles	Ames test	Chromosomal aberrations	Micronucleus assay	Comet assay	*In vivo* genotoxicity
SWCNT	○	●	●	●	●
MWCNT	○	○	●	●	●
TiO2	○	○	●	●	○
ZnO	○	●	○	●	○
Silver	○	●	●	●	●
Silica	○	●	●	●	●
Aluminium oxide	○	●	●	●	●
Iron oxide NPs	○	●	●	●	●
